# Alcohol-dose-dependent DNA methylation and expression in the nucleus accumbens identifies coordinated regulation of synaptic genes

**DOI:** 10.1038/tp.2016.266

**Published:** 2017-01-10

**Authors:** R Cervera-Juanes, L J Wilhelm, B Park, K A Grant, B Ferguson

**Affiliations:** 1 Department of Neurosciences, Oregon National Primate Research Center, Oregon Health and Science University, Beaverton, OR, USA; 2 Department of Public Health and Preventive Medicine, Oregon Health and Science University, Portland, OR, USA

## Abstract

Alterations in DNA methylation have been associated with alcohol exposure and proposed to contribute to continued alcohol use; however, the molecular mechanisms involved remain obscure. We investigated the escalating effects of alcohol use on DNA methylation, gene expression and predicted neural effects in the nucleus accumbens of rhesus macaques that self-administered 4% alcohol for over 12 months. Using an exploratory approach to identify CpG-rich regions, followed by bisulfite sequencing, the methylation levels of 2.7 million CpGs were compared between seven low-binge drinkers and nine heavy–very heavy drinking subjects. We identified 17 significant differential methylation regions (DMRs), including 14 with methylation levels that were correlated with average daily alcohol consumption. The size of the DMRs ranged from 29 to 158 bp (mean=63.7), included 4–19 CpGs per DMR (mean=8.06) and spanned a range of average methylation values from 5 to 34%. Eight of the DMRs mapped to genes implicated in modulating synaptic plasticity. Six of the synaptic genes have not previously been linked to alcohol use. Validation studies of these eight DMRs using bisulfite amplicon sequencing and an expanded set of 30 subjects confirmed the significant alcohol-dose-associated methylation of the DMRs. Expression analysis of three of the DMR-associated genes, *LRP5*, *GPR39* and *JAKMIP1*, revealed significant correlations between DMR methylation and whole-gene or alternative transcript expression, supporting a functional role in regulating gene expression. Together, these studies suggest that alcohol-associated synaptic remodeling may be regulated and coordinated at the level of DNA methylation.

## Introduction

Chronic and excessive alcohol use can lead to alcohol dependence, a relapsing and remitting condition that ultimately costs lives and disrupts families. In an effort to understand the neuroadaptive changes associated with dependent or compulsive drinking, investigations have focused on brain regions that process motivated behaviors and on cellular mechanisms underlying learning and memory. Accumulating evidence implicates the nucleus accumbens (NAc) core (NAcc) in the control of motivated behaviors by discrete cues.^1^ Thus, the NAcc can be viewed as a relay station selecting and integrating the most relevant environmental stimuli among competing limbic and cortical afferents to drive behavioral output,^2^ such as alcohol seeking. In the NAc, chronic alcohol use has been linked to changes in dendritic structures^3^ and neurotransmitter signaling^4^ thought to contribute to alcohol tolerance, craving and withdrawal.^5^ Thus, elucidating the molecular mechanisms that link alcohol use and these neural adaptations remains a challenge for fully understanding and treating alcohol dependence.

DNA methylation is an epigenetic mechanism that mediates the effects of the environment into altered chromatin structure, gene regulation and expression.^6^ Modified DNA methylation at individual loci has been linked to alcohol dependence,^7, 8^ and global CpG methylation has been reported to be higher in alcoholic populations.^9, 10, 11^ A recent genome-wide study identified differential DNA methylation in the prefrontal cortex from human post-mortem alcoholic and non-alcoholic subjects.^11^ A subset of the differentially methylated CpGs mapped to differentially expressed genes, suggesting that DNA methylation may be contributing to transcriptional regulation in alcoholics, although further studies are needed to verify this relationship. Nonetheless, the dependence of self-reported alcohol use in human studies severely limits the evaluation of alcohol dose effects on neural DNA methylation or associated changes in gene expression. In addition, unknown nicotine or drug use, or comorbid psychiatric and medical conditions have the potential to contribute confounding effects.

The nonhuman primate alcohol self-administration model provides an outstanding opportunity to discover both epigenetic and expression effects associated with alcohol use, overcoming the logistical constraints of human studies. In this model, macaques have 22 h per day access to 4% alcohol and water for a period of 12 months, enabling the collection of precise information of all alcohol consumed.^12^ In addition, macaques exhibit a natural broad distribution of drinking preferences similar to that of humans, enabling the study of varied alcohol use histories without relying on self-reported data. Furthermore, macaques born and raised in captivity typically have complete medical histories, and the immediate collection of tissues at the time of death provides optimal resources for epigenomic and transcriptomic studies. Finally, owing to the high conservation of genomic sequences, and similar neuroanatomy, the macaque offers high translational relevance for the study of alcohol-associated neuroadaptations.

In the present study, we leveraged the features of the macaque alcohol self-administration model to identify long-term alcohol dose effects on DNA methylation and associated gene expression in the NAcc. We combined the targeted selection of CpG-rich genomic regions, bisulfite sequencing and a statistical clustering approach to identify significant differential methylated regions (DMRs) among subjects that differed in their categorical alcohol consumption levels (low-binge (L/BD) and heavy–very heavy drinkers (H/VHD)). Both DNA methylation validation studies and associated gene expression analysis underscore the significant correlation between average daily alcohol dose, DNA methylation and gene expression. Eight of the genes identified map to synaptic genes, including *LRP5*, *GPR39* and *JAKMIP1*, encoding proteins modulating the balance between excitatory and inhibitory signaling.

## Materials and methods

### Subjects

Male rhesus macaques (*n*=30, *Macaca mulatta*), that were late adolescents, young adults and middle aged adults (4.3–4.9, 5.4–6.6 and 7.1–10.2 years at the start of the 12-month open access period, respectively; *n*=8, *n*=11 and *n*=11 per age group), were included in this study. All of the monkeys were born and reared at the Oregon National Primate Research Center (ONPRC) with their mothers until 2–3 years of age, and they were initially selected to minimize relatedness; the average kinship coefficient of all subjects was 0.003. Monkeys were individually housed, and all subjects underwent the same experimental conditions. Briefly, monkeys were allowed for visual, auditory and olfactory sensory contact with each other in a colony room with 12:12-h light–dark cycle with lights on at 0700 hours. All of the animal procedures used in this study were approved by the ONPRC IACUC and were performed in accordance with the NIH and the National Resource Council’s *Guide for the Care and Use of Laboratory Animals*.

### Drinking procedure

Voluntary and long-term ethanol self-administration was induced using schedule-induced polydipsia as previously described.^13^ Briefly, the monkeys were trained daily to use the operant panel and induced to drink 0.0, 0.5, 1.0 and 1.5 g kg^−1^ ethanol (4%) in 30 day epochs. During the following 12 months, subjects had open access (22 h per day) to 4% alcohol and water *ad libitum*. The alcohol intake data were collected and recorded in an automated manner by computer. Accumulative data on the alcohol self-administration consumption patterns in these macaques during the 12 months of open access have identified four stable and distinguishable categories, named low, binge, heavy and very heavy drinking. A combination of blood ethanol concentration (BEC), average g kg^−1^ per day consumption and percentage of days over a certain threshold have been identified as accurate parameters in distinguishing these four stable drinking patterns.^12^ Accordingly, subjects consuming >2 g kg^−1^ for more than 55% of the days were defined as binge drinkers (BDs). Those subjects consuming >3 g kg^−1^ for 20% of the days were classified as heavy drinkers (HDs), whereas the VHDs had more than 10% of the days with >4 g kg^−1^. Lastly, low drinkers (LDs) were those subjects that spent less than 55% consuming more than 2 g kg^−1^. Importantly, LD drinkers and BDs never or occasionally (respectively) reach BECs above 80 mg dl^−1^, the baseline measure of human intoxication.^12^ In contrast, HDs and VHDs routinely measure BECs above 80 mg dl^−1^. In the present study, the four categories were further combined into two groups based on propensitiy of the subjects to be intoxicated (BEC>80 mg dl^−1^). Thus, LDs and BDs were combined into L/BDs, whereas HDs and VHDs into H/VHDs.

This study included 17 L/BDs and 13 H/VHDs. Sixteen subjects (seven L/BDs and nine H/VHDs) were included in the genome-wide bisulfite sequencing study; the sample size was expanded to 30 subjects for the bisulfite amplicon sequencing (BSAS) validation study. On the basis of sample availability, 19 of the same animals were included in the RNA analysis (10 L/BDs and 9 H/VHDs).

### Tissue collection and genomic DNA isolation

After the 12-month open access period, and without imposed abstinence, a previously described, detailed necropsy protocol^14^ was used to systematically collect tissues from all subjects. Briefly, monkeys were anesthetized with ketamine (10 mg kg^−1^), maintained on isoflurane and perfused with ice-cold oxygenated monkey perfusion solution (containing (in mM) 124 NaCl, 23 NaHCO_3_, 3 NaH_2_PO_4_, 5 KCl, 2 MgSO_4_, 10 D-glucose, 2 CaCl_2_). Brains were quickly removed and sectioned along the coronal plane using a brain matrix.^15^The block containing the NAcc was initially selected by each individual’s magnetic resonance imaging and verified using visible landmarks. In macaques, the NAcc is ~2 mm × 2 mm and extends ~3 mm rostral/caudal.^16^ The core is differentiated from the shell based on visible landmarks. Using the curvature of the internal capsule, the area just ventral to its end is the NAcc. From the frozen 4 mm coronal brain block maintained on dry ice, a small circular dissection of ~1 mm^3^ was made, taking care to not collect white matter from the tract (dorsal to the core). This relatively small dissection avoids the NAc shell and yields enough tissue for nucleic acid isolation. Genomic DNA and RNA were extracted from the NAcc using the All Prep DNA/RNA/microRNA Universal Kit (Qiagen Sciences, Germantown, MD, USA) following the manufacturer’s recommendations.

### High-throughput DNA methylation analysis

Three micrograms of genomic DNA were sheared using a Bioruptor UCD200 (Diagenode, Denville, NJ, USA), generating fragments ~180 bp. The SureSelect XT Human Methyl-Seq library preparation (Agilent Technologies, Santa Clara, CA, USA) was used following the manufacturer’s instructions. The libraries were then bisulfite-treated using EZ DNA Methylation-Gold (Zymo Research, Irvine, CA, USA), and quantified using a 2100 Bioanalyzer (Agilent Technologies). DNA libraries were sequenced on an Illumina HiSeq2500 at the OHSU Massively Parallel Shared Sequence Resource.

### CpG methylation rate analysis

The quality of the bisulfite-converted sequencing reads was assessed with FastQC. Reads were trimmed and aligned to the rhesus macaque reference genome (MacaM^17^), and then the bisulfite conversion rates were evaluated, insuring all libraries were >98% converted, and CpG methylation was evaluated using Bismark.^18^ The methylation rates were calculated as the ratio of methylated reads over the total number of reads. Methylation rates for CpGs with fewer than 10 reads were excluded from further analysis. The remaining CpGs (2.7 million) had an average of 60 × read coverage. All sequence reads were submitted to the Sequence Read Archive at NCBI under project accession number PRJNA294610. An overview of these results is described in Supplementary Table 1. The differential analysis of the CpG methylation levels is described in Statistical analysis below.

### Bisulfite amplicon sequencing

Candidate DMR methylation levels were validated using targeted BSAS. Primers were designed within 200 bp of each DMR, using the Bisulfite Primer Seeker tool from Zymo Research (Supplementary Table 2). Each gDNA (250 ng) was bisulfite-converted using EZ DNA Methylation-Gold (Zymo Research) and 12.5 ng of bisulfite-converted DNA was used for each polymerase chain reaction (PCR). Library construction, analysis of the percent methylation at each CpG in each amplicon and PCR allele bias correction were performed as previously described.^19^

### High-throughput real-time PCR

RNA extracted from the same NAcc tissues was used for quantitative reverse transcriptase-PCR (RT-PCR) analysis. The Fluidigm Reverse Transcription Master Mix (Fluidigm, San Francisco, CA, USA) was used to reverse-transcribe 100 ng of each RNA sample following the manufacturer’s instructions. Next, the complementary DNA was pre-amplified, and unincorporated primers were removed following the manufacturer’s instructions. The reactions were diluted (10 × ) with 43 μl of TE buffer (TEKnova, Hollister, CA, USA).

qPCR was performed using the BioMark HD System and the 96.96 GE Dynamic Arrays (Fluidigm) in triplicate assays. The Fluidigm sample premix and the assay premix were prepared following the manufacturer’s instructions. The samples and reagents were mixed using the Nanoflex IFC controller (Fluidigm). Thermal qPCR conditions were as follows: 95 °C for 60 s, 35 cycles of 95 °C for 5 s and 60 °C for 20 s. Data were processed by automatic threshold for each assay, with derivative baseline correction using the BioMark Real-Time PCR Analysis Software 3.1.2 (Fluidigm). The quality threshold was set at the default of 0.65.

The primer sequences are listed in Supplementary Table 3.

The mRNA expression levels were normalized as previously described,^19^ except for using the geometric mean of three constitutive genes: *B-Actin*, *Tubulin1* and *Phosphoglycerate kinase (PGK1* (ref. 20)). We also confirmed that different levels of alcohol use did not affect their expression in the NAcc (data not shown).

### Statistical analysis

The exploratory nature of the present study limited our ability to estimate *a priori* the sample size needed to detect the effects of a broad range of alcohol on the methylome. Thus, we used a group-comparison (L/BD versus H/VHD) that was previously established, based on the specific drinking parameters during 12 months of alcohol consumption, to maximize the opportunity to detect significant effects of alcohol dose on DNA methylation levels.

One of the limitations of the analysis of genome-wide bisulfite sequencing data is that the significance of the individual CpG sites may be dampened after multiple-testing correction on potentially millions of sites. Thus, we employed the comb-p method that combines *P*-values in sliding windows and accounts for spatial correlations across the genome.^21^ In detail, the CpG methylation rates of L/BD (*n*=7) and H/VHD (*n*=9) subjects were first subjected to the Wilcoxon two-sample Independent test using the wilcox.test function in the R programming language.^22^ The comb-p method uses a sliding window correction where each Wilcoxon *P*-value is adjusted by applying the Stouffer–Liptak–Kechris (slk) method^23, 24, 25^ of neighboring *P*-values as weighted according to the observed autocorrelation (ACF) at the appropriate lag. Briefly, comb-p first calculates the ACF at varying distance lags, and then the ACF is used to perform the slk correction where each *P*-value is adjusted according to adjacent *P*-values as weighted according to the ACF. Thus, a given *P*-value will be pulled lower if its neighbors also have low *P*-values and likely remain insignificant if the neighboring *P*-values are also high. Next, a *q*-value score based on the Benjamini–Hochberg false discovery rate correction is calculated. The peak-finding algorithm is used to find enrichment regions. Once the regions are identified, a *P*-value for each region can be assigned using the Stouffer–Liptak correction. Then, the false discovery rate *q*-value is used to define the extent of the region, whereas the slk-corrected *P*-value and a one-step Sidak multiple-testing correction^26^ is used to define the significance of the region. Parameters for Comb-p were DIST=300, STEP=60 and THRESHOLD=0.05.

The Shapiro–Wilk test (appropriate for small sample sizes) was used to assess the normality of the average methylation and single CpG methylation rates for the eight amplicons analyzed by BSAS, and also the mRNA expression for *LRP5*, *GPR39* and *JAKMIP1*. The variables analyzed followed a normal distribution. We used the 1.5xIQ (interquartile range) method to identify outliers, which were subsequently excluded from the corresponding analysis. The sample size for each analysis is specified in the Results section as well as in the figure legends.

Before applying Independent *t*-test to compare the BSAS average methylation rates and mRNA expression between L/BDs and H/VHDs, the Levene’s test was used to test homogeneous variance assumption for the *t*-test (p*ARHGEF7*-DMR=0.099, p*CDH5*-DMR=0.028, p*JAKMIP1*-DMR=0.050, p*KIRREL3*-DMR=0.676, p*GPR39*-DMR=0.177, p*NTM*-DMR=0.064, p*LRP5*-DMR=0.309, p*NBEA*-DMR=0.302, p*JAKMIP1-*exon 1B-mRNA=0.021, p*JAKMIP1-*exon 1A-mRNA=0.921, pGPR39-mRNA=0.145, p*LRP5*-mRNA=0.207). When the variance was heterogeneous (*CDH5*-DMR, *JAKMIP1-*exon 1B-mRNA), the Welch–Satterthwaite method was used for estimating the s.e.

Pearson correlation analysis was used to explore associations between mRNA expression, DMR average methylation and average ethanol (g kg^−1^ per day) consumption.

All statistical analyses were carried out using IBM SPSS Statistics (Armonk, NY, USA), R and comb-p software, with values *α*<0.05.

## Results

### Description of the subjects and their ethanol-drinking patterns

Self-reported data indicate that human subjects with alcohol use disorders consume a wide range of alcohol, from 0.7 to >4 g kg^−1^ per day.^11, 27, 28, 29^ Similarly, the macaques in this study consumed between an average of 0.5 and 4.4 g kg^−1^ per day during the 12 months of open access to ethanol and water (Supplementary Figure 1b). The subjects were classified as H/VHD if they had more than 20% of days with 3 g kg^−1^ or more ethanol consumption, and L/BD if they did not exceed the 20% threshold.^12^ The mean daily alcohol use was 1.9 and 3.2 g kg^−1^ per day for L/BD and H/VHD, respectively. In addition, we note that the L/BD group very rarely or occasionally reached BECs of 80 mg dl^−1^, a measure of alcohol intoxication, whereas the H/VHD members regularly exceeded the 80 mg dl^−1^ threshold. We previously confirmed that the amount of ethanol consumed did not reflect general differences in thirst and were not associated with the age of the subjects.^19^

### Alcohol-associated differentially methylated CpGs in the NAcc

Using an enrichment approach targeting GENCODE promoters,^30^ CpG islands (CGI), shores and shelves, and DNase I-hypersensitive sites located in or near RefGenes, followed by bisulfite sequencing, we analyzed roughly 3.1 million CpGs per subject. Of these, 2.7 million CpGs per individual met our quality requirements (Supplementary Table 1). In all, 74 032 CpGs (2.7%) had significant methylation differences between L/BD and H/VHD subjects (Wilcoxon test). The significant CpGs showed a greater proportion of hypermethylated CpGs in H/VHDs (51 500/74 032=70% Supplementary Figure 2).

### Alcohol-associated DMRs in the NAcc

**Figure 1:**
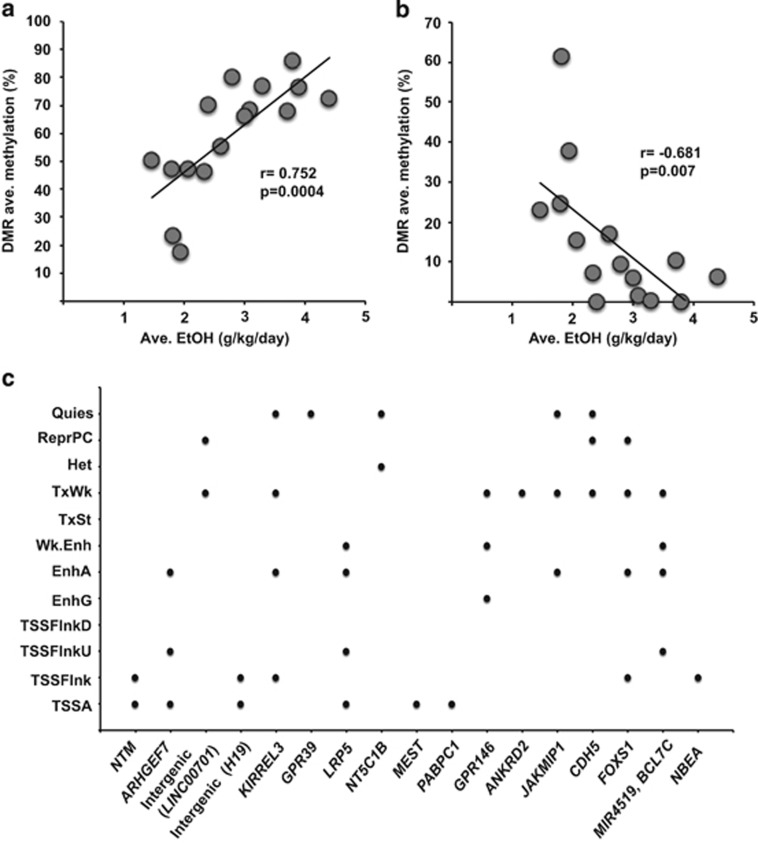
Alcohol-associated DMRs identified in the rhesus macaque NAcc. (**a**, **b**) Correlation between the average methylation of 15 hypermethylated or 2 hypomethylated DMRs and daily average ethanol consumed across 16 and 15 male rhesus macaques, respectively. (**c**) Distribution of chromatin states associated with the 17 NAcc DMRs, based on seven brain regions reported in the Epigenomics Roadmap Database.^33^ DMR, differentially methylated region; EnhA, active enhancer; EnhG, genic enhancer; Het, associated heterochromatin; Quies, quiescent state; NAcc, nucleus accumbens core; ReproPC, repressed polycomb region; TSSA, active transcription start site; TSSFlank, TSS flanking sequence; TSSFlnkU and TSSFlankD, upstream and downstream TSS flanking sequence, respectively; TxSt, strong transcriptional activity; TxWk, weak transcriptional activity; Wk.Enh, weak enhancer.

Although the methylation of a single CpG can influence gene expression,^31^ it is challenging to pinpoint functional methylation changes among the thousands of significant CpGs detected. An alternative approach is to identify contiguous differentially methylated CpGs, which minimizes false discovery and enhances the prospect of identifying functional effects.^21, 32^ In this study, we applied the comb-p method,^21^ which identifies regionally correlated *P*-values, applies a false discovery rate correction to define the extent of the region and a one-step Sidak multiple-testing correction^26^ to define the significance of the DMR. This approach identified a set of 17 DMRs distinguishing L/BDs and H/VHDs (Table 1). The size of the DMRs ranged from 29 to 158 bp (mean=63.7), included 4–19 CpGs per DMR (mean=8.06) and had an average CpG density of 0.13. Neighboring CpGs within each DMR exhibited concordant DNA methylation differences. Whereas alcohol consumption was generally associated with higher DMR methylation (Table 1), 2 of the 17 DMRs were hypomethylated among H/VHDs (DMRs linked to *NTM* and *LRP5*; Table 1). The global CpG methylation within these two DMRs was negatively correlated with the daily average amount of alcohol consumed (seven L/BD and eight H/VHD, one subject had no data for these two DMRs; *r*=−0.681, *P*=0.007; Figure 1b). Alternatively, the global average CpG methylation within the remaining 15 DMRs (seven L/BD and nine H/VHD) was positively associated with the daily average amount of ethanol consumed by the 16 subjects included in this study (*r*=0.752, *P*=0.0004; Figure 1a). Overall, 14 of the DMRs had average CpG methylation levels that were significantly correlated with average g kg^−1^ per day ethanol (Table 1), suggesting that DNA methylation of these regions is dynamically modified by the amount of alcohol consumed.

To clarify the potential functional roles of these DMRs, we first considered the location of DMRs relative to CGI.^34^ The DMRs were located within eight CGIs, four CGI shores or shelves and five CpG open sea regions (Table 1). Next, we determined the DMR genomic context, including location within the gene body, promoter (up to 5 kb upstream of the transcription start site, TSS) or intergenic (excluding promoters) regions. As gene annotations in the rhesus macaque genome are currently incomplete, each DMR was mapped to the orthologous human gene. Human-macaque DMR sequence conservation ranged from 90 to 97% (data not shown). Fifteen of the DMRs were located within a gene body (Table 1). In addition, 13 of these genes encode multiple alternative transcript variants (TVs), and 12 of the DMRs are located within 10 kb of an alternative first exon (data not shown). We then used the Epigenomics Roadmap database to compare the chromatin states overlapping with the DMRs.^33^ We only considered the chromatin states measured in the seven human brain regions reported (hippocampus middle, substantia nigra, anterior caudate, cingulate and angular gyrus, inferior temporal lobe, dorsolateral prefrontal cortex); NAcc analysis is not included in the database. The DMRs can overlap with multiple chromatin states, and these analyses predict that five DMRs coincide with TSS, and nine overlap TSS, enhancer and predicted active transcriptional regions (Figure 1c). Although the rhesus macaque and human DMR sequence identity is less than 100% (ranging from 90 to 97%), the CGI and genomic context data suggest that the DMRs are associated with gene regulatory functions.

### Alcohol-associated DMRs linked to genes with synaptic functions

**Figure 2:**
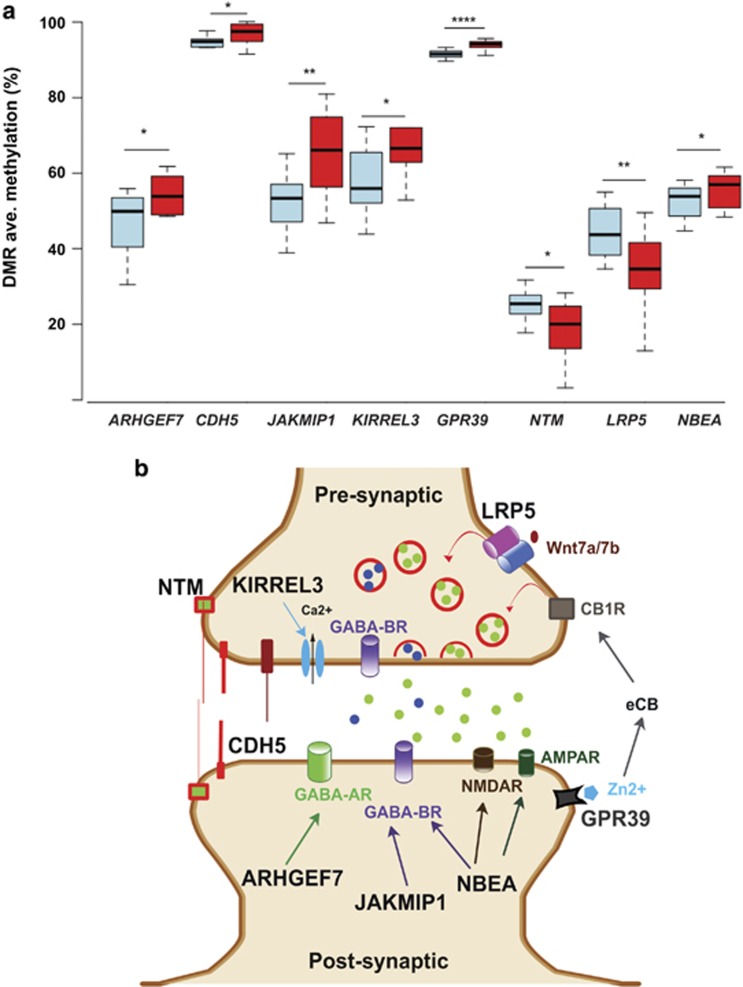
Eight DMRs are associated with genes with synaptic functions. (**a**) The DMR average methylation levels among 17 L/BD (blue) or 13 H/VHD (red) subjects per each DMR-associated gene (independent *t*-test). *P<0.05, **P⩽0.01, ****P⩽0.0001. (**b**) Graphic model of the presynaptic and post-synaptic contexts of eight DMR-linked gene products, independent of the neuron type. The genes identified by the DMRs are indicated in black. Narrow arrows indicate interactions between DMR-implicated proteins and other synaptic proteins. Neurotransmitters and signaling molecules are represented by colored circles (GABA in dark blue; Glutamate in green; Wnt7a/7b in brown; Zn^2+^ in light blue). Synaptic vesicles are indicated on the presynaptic terminus as red circles containing neurotransmitters. Ca^2+^ channels are shown as blue ovals. Receptors are indicated by cylinders (GABA_B_, GABA_A_, NMDAR, AMPAR and LRP5). CB1R is the cannabinoid receptor 1. eCB, endocannabinoids; DMR, differentially methylated region; H/VHD, heavy/very heavy drinker; L/BD, low/binge drinker.

The 17 DMRs mapped to genes encoding microRNAs and noncoding RNAs (ncRNAs) (*MIR4519*, *H19*), transcription and translation regulators (*ANKRD2*, *PABPC1*, *FOXS1*, *NT5C1B*), cell surface receptors (*LRP5*, *GPR39*), cell adhesion molecules (*CDH5*, *NTM*, *KIRREL3*), protein trafficking (*ARHGEF7*, *JAKMIP1*, *NBEA*) and regulatory molecules (*MEST*; Table 1). Eight of the DMRs mapped to genes regulating synaptic plasticity, a mechanism known to be altered in alcohol dependence. In detail, genes were implicated in modulating dendritic spine dynamics, neurotransmitter release and receptor trafficking or stabilization (Figure 2b). Owing to their potential role in coordinating alcohol-induced effects on synaptic plasticity, these eight DMRs were selected for validation studies. Using BSAS and an expanded number of subjects totaling 17  L/BDs and 13 H/VHDs, we confirmed a significant DNA methylation difference between L/BD and H/VHD subjects in all eight DMRs, and alcohol-dose-correlated methylation in seven of the DMRs (Figure 2a; Supplementary Table 4). There was no effect of the age on differential methylation levels for the eight DMRs analyzed (one-way analysis of variance, *P*>0.05).

We next investigated the possible functional effects of the synaptic gene-associated DMRs. All eight DMRs are located in the gene body of synaptic genes encoding multiple transcripts. Six DMRs overlap with chromatin states predicted to function as TSS (including flanking regions) or enhancers, whereas the remaining two (*CDH5* and *GPR39*) are associated with low transcriptional activity regions (Figure 1c). We selected three DMRs (*LRP5*, *GPR39* and *JAKMIP1*) for gene expression analysis based of their roles in regulating glutamate and gamma-aminobutyric acid (GABA) signaling, two of the neurotransmitter systems altered by alcohol use. In addition, the DMR location and gene structure in each case suggested different regulatory functions. As differential DNA methylation in proximal/distal enhancer regions can serve to modulate gene expression and alternative splicing,^35^ we evaluated both total gene and TV expression for these three DMR-linked genes. In every case, there was no effect of the age of the subjects on the expression levels of these three genes (one-way analysis of variance, *P*>0.05).

**Figure 3:**
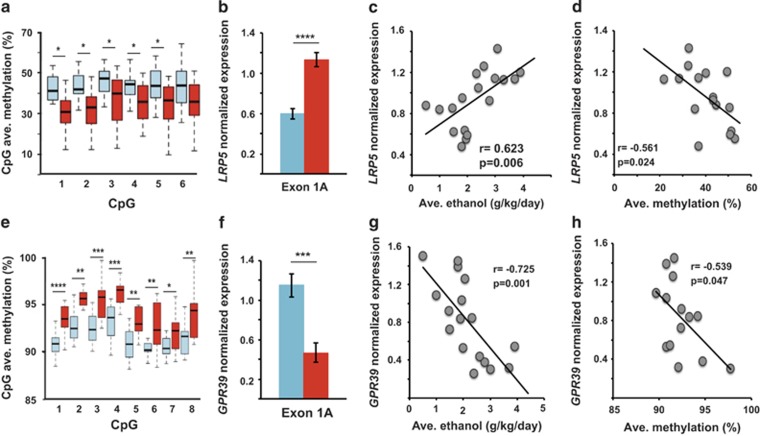
Characterization of DNA methylation and gene expression associated with *LRP5* and *GPR39*. Blue indicates L/BD and red indicates H/VHD. (**a**) *LPR5*-DMR single CpG methylation levels among L/BD and H/VHD macaques (*n*=30, Independent *t*-test). (**b**) *LRP5* TV-201 relative expression in L/BD and H/VHD macaques (*n*=19, Independent *t*-test). (**c**) Correlation between *LRP5* TV-201 expression and average daily ethanol consumption (*n*=18). (**d**) Correlation between *LRP5* TV-201 relative expression and DMR average methylation (*n*=16). (**e**) *GPR39*-DMR single CpG methylation levels among L/BD and H/VHD macaques (*n*=30, Independent *t*-test). (**f**) *GPR39* TV-201 relative expression in L/BD and H/VHD macaques (*n*=19, Independent *t*-test). (**g**) Correlation between *GPR39* TV-201 expression and average daily ethanol consumption (*n*=17). (**h**) Correlation between *GPR39* TV-201 expression and DMR average methylation (*n*=14). *P<0.05, **P⩽0.01, ***P⩽0.001, ****P⩽0.0001. Error bars are mean±s.e.m. DMR, differentially methylated region; H/VHD, heavy/very heavy drinker; L/BD, low/binge drinker.

We first investigated the alcohol-dose-associated gene expression of *LRP5*, using the same group comparisons as used for the methylation studies. The DMR associated with the *LRP5* gene is located within intron 5, ~75 kb downstream of the first exon (exon 1 A) of macaque TV-201 and -202 (Ensembl MMUL 1.0 (ref. 36)). The *LRP5*-DMR CpGs were hypomethylated among H/VHDs (Figure 3a), and the average methylation level was negatively correlated with alcohol consumption level (*r*=−0.700, *P*=0.004; Table 1). Consistent with DMR hypomethylation, the expression of exon 1A was higher in H/VHDs (Figure 3b) and was positively correlated with the amount of alcohol consumed (*r*=0.623, *P*=0.006; Figure 3c). The DMR methylation level was negatively correlated with exon 1A expression (*r*=−0.561, *P*=0.024; Figure 3d), suggesting a functional role in regulating transcription. Because the exons in TV-203 are within the other two TVs, we were unable to measure the expression of exon 1B independently. However, the very similar increase in expression of exon 1A and 1B measured together suggests that there is either no expression or a parallel increase in exon 1B expression as well (data not shown). Transcription factors AP-2α (activating enhancer-binding protein 2) and GATA-3 are predicted to bind to the DMR (TRANSFAC^37^).

The *GPR39* gene has a DMR located within the unique exon (exon 1A) encoding macaque TV-201. To ensure the detection of exon 1A in the macaques, we designed primers to exclusively amplify exon 1A. The DMR is ~0.7 kb downstream of the TSS. In this case, the *GPR39*-DMR was hypermethylated in the H/VHDs (Figure 3e), and DMR methylation was positively correlated with alcohol consumption (*r*=0.565, *P*=0.028; Table 1). The higher methylation in H/VHDs was associated with lower expression of TV-201 (Figure 3f) and expression was negatively correlated with the amount of alcohol consumed (*r*=−0.725, *P*=0.001; Figure 3g). As with *LRP5*, *GPR39* expression was inversely correlated with DMR methylation levels (*r*=−0.539, *P*=0.047; Figure 3h). The transcription factor AP-2α is predicted to bind to this DMR (TRANSFAC^37^).

**Figure 4:**
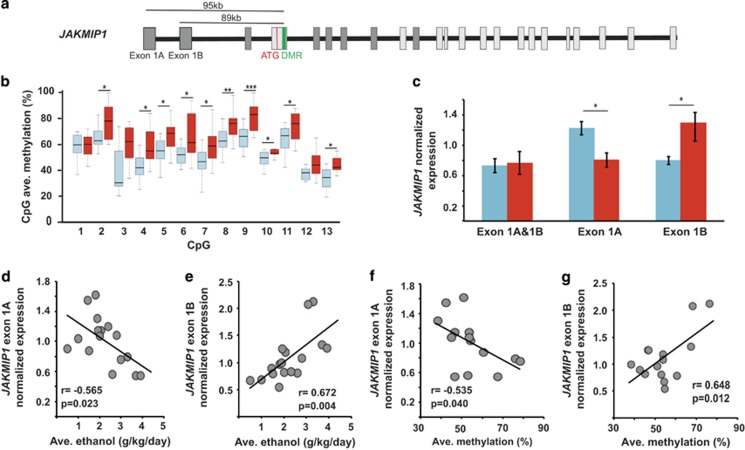
Summary of DMR location and gene structure, CpG methylation and transcript expression for *JAKMIP1*. L/BD is shown in blue and H/VHD is shown in red. (**a**) *JAKMIP1* gene structure. The DMR is indicated by a green block, and exons by gray boxes. The exons common to all TVs are represented in dark gray, whereas those specific to certain TVs are in light gray. Distance between alternative exon 1A or 1B and the DMR is shown above gene. (**b**) CpG methylation levels for L/BD and H/VHD subjects (*n*=30, Independent *t*-test). (**c**) Relative expression of *JAKMIP1* of both exon 1A and exon 1B containing transcripts either combined (Exon 1A and 1B) or measured individually in L/BD and H/VHD subjects (Independent *t*-test). Correlation between JAKMIP1-exon 1A (**d**, *n*=16) or exon 1B (**e**, *n*=17) expression and average daily ethanol consumption (*n*=19). Correlation between JAKMIP1-exon 1A (**f**, *n*=14) or exon 1B (**g**, *n*=14) expression and average DMR methylation level. *P<0.05, **P⩽0.01, ***P⩽0.001. Error bars are mean±s.e.m. DMR, differentially methylated region; H/VHD, heavy/very heavy drinker; L/BD, low/binge drinker; TV, transcript variant.

*JAKMIP1* uses two alternative first exons to encode three different protein-coding TVs in macaques (TV-201, -202 and -203, Ensembl MMUL 1.0 (ref. 36)). The DMR associated with the *JAKMIP1* gene is located within alternative exon 3, and 89–95 kb downstream of the two alternative first exons (termed 1A and 1B; Figure 4a). The *JAKMIP1*-DMR is hypermethylated in H/VHDs (Figure 4b) and is positively correlated with the amount of alcohol consumed (*r*=0.595, *P*=0.032; Table 1). The expression of TVs that contain exon 3 was not different from TVs in which exon 3 is absent, suggesting that the DMR does not regulate alternative splicing of exon 3 (L/BD_exon3_=0.82 versus H/VHD_exon3_=1.13, *P*=0.032; L/BD_no_exon3_=0.98 versus H/VHD_no_exon3_=1.3, *P*=0.006). Moreover, the ENCODE chromatin states^33^ in brain tissues indicate that the DMR overlaps with an active enhancer domain (Figure 1c), suggesting that the DMR may regulate the expression of the alternative first exons by modulating enhancer activity. Multiple predicted binding sites for AP-2α (TRANSFAC^37^) are present within this DMR. Thus, we investigated the expression level of the TVs that include either exon 1A or exon 1B (Ensembl MMUL 1.0 (ref. 36)). Interestingly, we detected both a significant reduction in exon 1A expression, and an increase in exon 1B expression in H/VHDs (Figure 4c). In addition, the expression levels of each alternative first exon were significantly correlated with the amount of alcohol consumed (exon 1A: *r*=−0.565, *P*=0.023; exon 1B: *r*=0.672, *P*=0.004; Figure 4d, e) and the DMR methylation levels (exon 1A: *r*=−0.535, *P*=0.040; exon 1B: *r*=0.648, *P*=0.012; Figure 4f, g).

## Discussion

The present study reports use of the macaque alcohol self-administration model to identify alcohol-dose-associated DNA methylation in the NAcc. By focusing on clusters of differentially methylated CpGs in two alcohol-dose groups (L/BD and H/VHD), we identified DMRs linked to genes of high relevance to alcohol use using a relatively small number of subjects. Underscoring the power of this approach, the significant differential methylation of all eight synaptic gene DMRs was validated using a larger sample set, and alcohol-dose-associated methylation was identified in seven DMRs. Moreover, the analysis of three of the genes suggests that the alcohol-dose-dependent expression of TVs is coordinately regulated at the level of DNA methylation.

Synaptic remodeling, including dendritic spine structural dynamics, has been proposed to contribute to alcohol craving and addiction.^38^ Three of the DMRs identified are linked to genes (*NTM, CDH5, KIRREL3)* implicated in dendritic spine remodeling^39, 40, 41^ processes known to be altered in the NAc of rodents^42, 43^ and in the putamen of macaques^44^ after alcohol use.

Chronic alcohol use can also induce lasting changes in activity-dependent synaptic plasticity by altering the balance between excitatory and inhibitory neurotransmission. These changes are mediated by altering neurotransmitter release and receptor composition and function.^4^ Among the DMR-linked genes identified, two are implicated in controlling neurotransmitter release (*LRP5* and *GPR39*) and three contribute to neurotransmitter receptor trafficking and abundance (*JAKMIP1, ARHGEF7, NBEA*).

The *LRP5* gene, encoding the low-density lipoprotein receptor-related protein 5, functions as a presynaptic co-receptor for Wnt in the Wnt-*β*-canonical pathway. Extensive evidence supports a critical role of the Wnt-*β*-canonical pathway in regulating synaptic plasticity, including the accumulation of synaptic proteins, the formation of the active zones, stimulation of recycling and exocytosis of synaptic vesicles and modulation of trafficking of receptors.^45^ Our study identifies a direct relationship between heavy alcohol consumption, decreased *LRP5*-DMR methylation and increased *LRP5* expression. We postulate that overexpression of *LRP5* could facilitate Wnt signaling, promoting increased neurotransmitter release.

The present study also identified significant associations between alcohol consumption, increased DMR methylation and decreased *GPR39* expression. *GPR39,* which encodes Zn^2+^-binding G-coupled protein receptor 39, has not previously been linked to alcohol use. However, recent evidence indicates that Zn^2+^ binding to *GPR39* promotes endocannabinoid release,^46^ which in turn modulates presynaptic neurotransmitter release through the CB1 receptor.^47^ Several lines of evidence support a link between Zn^2+^, endocannabinoids and alcohol use disorders. Clinical studies have revealed that Zn^2+^ deficiency is common among alcoholics,^48^ and numerous studies have reported significant alterations in the endocannabinoid system following chronic ethanol consumption.^49, 50^ In addition, Zn^2+^ modulates alcohol-sensitive targets, including GABA_A_ and GABA_B_, N-methyl-d-aspartate, AMPA and glycine receptors.^51^ Our finding that heavy alcohol use is associated with decreased *GPR39* expression predicts a role in downregulating the inhibitory endocannabinoid pathway, facilitating glutamate release^52^ and, to a lesser extent, GABA release at GABAergic synapses.^53^

The identification of direct association between alcohol dose and DNA methylation and gene expression of *JAKMIP1* (janus kinase and microtubule interacting protein 1) also suggests its role in promoting synaptic adaptation. JAKMIP1 regulates GABA_B_ signaling by mediating GABA-_B_R1 trafficking to the cell membrane.^54^ In addition, *JAKMIP1* functions as an RNA-binding protein that regulates translation of GABA-_B_R2. GABA-_B_Rs mediate the slow and prolonged phase of synaptic inhibition,^54^ and have been implicated in the rewarding effects of drugs of abuse.^55^ GABA-_B_ agonists decrease alcohol consumption and craving in humans and severity of alcohol-withdrawal symptoms in humans and rats.^56^ Consistent with this, *GABA-*_*B*_*R1* expression is decreased in the hippocampus of alcoholics.^55^ Our findings of alcohol-dose-associated shift in *JAKMIP1* TV expression implicate it as an additional mechanism that may modulate GABA_B_ signaling, by regulating the translation and trafficking of GABA-_B_R to the cell surface.

Overall, the methylation and expression data are consistent with studies demonstrating a shift in the balance of excitatory/inhibitory transmission that biases the circuit toward an enduring increase in synaptic activation. For instance, elevations in extracellular glutamate and alterations in GABAergic signaling are observed after chronic alcohol use.^4, 44, 53^ Our results identify *LRP5* and *GPR39* as facilitators of glutamate signaling in an alcohol-dose-dependent manner. In addition to *JAKMIP1*, we identified two other genes modulating the balance between excitatory and inhibitory signaling. Specifically, *NBEA* regulates N-methyl-d-aspartate receptor and GABA-_A_R trafficking,^57^ whereas *ARHGEF7* modulates GABA-_A_R membrane clustering.^58^ Thus, we postulate that *JAKMIP1, NBEA* and *ARHGEF7* enhance excitation among H/VHDs by modulating GABA signaling. The methylation levels of all five genes (*LRP5, GPR39*, *JAKMIP1*, *NBEA* and *ARHGEF7*) are correlated with alcohol consumption, suggesting that these genes may coordinately shift the balance between excitatory and inhibitory signaling in a dose-dependent manner. Further studies are needed to clarify the relationship between these genes and alterations in excitatory and inhibitory signaling pathways.

In summary, the DMRs identified in this study provide novel insight into the role of DNA methylation in regulating alcohol-dose-dependent gene expression in the primate brain. Studies are currently underway to understand how DNA methylation contributes to the regulation of alternative TVs. Our discovery of alcohol-associated DNA methylation signals in the NAcc of rhesus macaques is consistent with similar methylation findings in the same genes (*LRP5* and *NTM)* in the prefrontal cortex of alcoholics,^11^ underscoring the relevance of the macaque alcohol model. DMRs detected in six other synaptic genes not previously linked to alcohol use clarify molecular mechanisms promoting alcohol-associated synaptic adaptations that may be specific to the NAcc. Future studies addressing the specific synaptic adaptation mechanisms occurring in different brain areas will be needed to fully understand the overall process of alcohol dependence. Our findings, in addition to implicating modulators of DNA methylation as a treatment for compulsive alcohol seeking, offer new individual targets to test for the treatment of alcohol dependence. Although our data focus on the role of DMRs in coordinating alcohol-dose-dependent neuroadaptation, we anticipate that there are also single CpGs that serve similar functions. Future studies will be needed for the identification of such regulatory CpGs. The CpG methylation states detected in our study provide a snapshot of the DNA state following 12 months of alcohol use. It is not known whether the DMRs were induced by alcohol, or rather were pre-existing epigenetic liabilities that influenced alcohol consumption. Future studies aimed at identifying pre-existing epigenetic marks and longitudinal study designs will be needed to identify a causative role between alcohol use and DNA methylation. In addition, functional tests using pharmacological or gene manipulation approaches will be crucial in determining the role of the DMR methylation and gene expression findings in escalating alcohol use and dependence.^59^

## Figures and Tables

**Table 1:** DMRs associated with alcohol dose

*Chromosome position*	*Genes within 50 kb*	*Genomic context*	*CGI context*	*# CpGs*	*Ave methylation Δ (%)*	*Sidak* P*-value*	*Methylation versus ethanol correlation*	
							*r*	*P-value*
chr10:92943378-92943534	***ANKRD2**, HOGA1, C10orf62, MORN4, UBTD1*	Gene body exon 3	Island	19	13.79	4.52E−08	0.562	**0.0230**
chr12:10943829-10943898	***BCL7C**, MIR762HG, MIR762, CTF1, MIR4519, CTF2P, FBXL19, FBXL19-AS1*	Gene body exon 3	Shore	9	19.41	2.44E−05	0.566	**0.0220**
chr13:92561716-92561755	***ARHGEF7***	Gene body intron 7	Open sea	5	28.39	8.60E−04	0.539	**0.0380**
chr11:1814166-1814220	*MRPL23, H19, MIR675, LINC01219, MRPL23-AS1*	Intergenic	Shelf	4	24.32	4.28E−04	0.835	**0.0002**
chr16:50552282-50552347	***CDH5**, LOC105371318, LINC00920, BEAN1*	Gene body exon 12	Island	11	10.08	5.95E−04	0.308	0.2850
chr04:6299173-6299257	***JAKMIP1***	Gene body exon 3	Island	13	17	3.30E−03	0.595	**0.0320**
chr11:118754778-118754843	***KIRREL3**, LOC105369561, LOC101929427*	Gene body intron 2	Open sea	8	10.89	4.17E−03	0.394	0.1460
chr07:34906263-34906329	***C7orf50**, **GPR146**, MIR339, LOC105375120, GPER1*	Gene body intron 2 (***C7orf50)***/ intron 1 ***(GPR146)***	Shore	7	9.49	9.84E−03	0.134	0.6630
chr16:71325524-71325565	*LINC00701, PFKP*	Intergenic	Open sea	6	10.17	9.48E−03	0.853	**0.0004**
chr15:32231385-32231448	***FOXS1**, MYLK2, DUSP15, TTLL9, TPX2*	Gene body exon 1	Island	7	14.48	4.85E−03	0.654	**0.0080**
chr02a:18599090-18599139	***NT5C1B**, NT5C1B-RDH14, LOC105373456, RDH14*	Gene body exon 5	Island	9	19.83	1.80E−03	0.584	**0.0460**
chr13:4768685-4768746	***PABPC1**, LOC105375673, MIR7705*	Gene body exon 2	Island	7	22.99	1.33E−03	0.812	**0.0020**
chr07:156179985-156180038	***MEST**, MESTIT1, MIR335, COPG2, COPG2IT1, LOC105375505, CEP41*	Gene body exon 1C	Island	8	9.08	1.20E−03	0.75	**0.0010**
chr02b:17817906-17817960	***GPR39***	Gene body exon 1	Island	8	4.94	1.10E−02	0.565	**0.0280**
chr11:123302781-123302825	***NTM***	Gene body intron 1	Open sea	5	−16.26	1.71E−02	−0.675	**0.0080**
chr11:6187519-6187578	***LRP5**, LOC101928175*	Gene body intron 5	Open sea	6	−12.83	1.94E−02	−0.7	**0.0040**
chr13:14974434-14974463	***NBEA**, MAB21L1*	Gene body intron 40	Shore	5	9.1	3.56E−02	0.729	**0.0010**

Abbreviations: CGI, CpG island; DMR, differentially methylated region; TSS, transcription start site.

Detail for each DMR: chromosome position in the MacaM genome, genes located within 50 kb of the DMR (closest gene shown in bold; using GRCh37/hg19 genome), genomic context (gene body: intron or exon; promoter: up to 5 kb of TSS; using GRCh37/hg19 genome), CGI context, the number of CpGs within the DMR, the average methylation difference between the comparison classes, corrected Sidak *P*-value for the DMR and the Pearson’s correlation coefficient and *P*-value between DMR average methylation level and the average g kg^−1^ per day ethanol consumed (*P*-values < 0.05 are shown in bold).
