# Supplementary material for: Alcohol-dose-dependent DNA methylation and expression in the nucleus accumbens identifies coordinated regulation of synaptic genes
Source: Transl Psychiatry. 2017 Jan 10;7(1):e994–. doi: 10.1038/tp.2016.266 (PMC5545731; doi:10.1038/tp.2016.266)
Supplement: Supplementary Table 1 [file tp2016266x1.docx]

**Supplementary table 1**. Summary of the sequence data generated for each subject.

| **Sample ID** | **Group** | **Reads** | **Gigabases** | **Mapping Efficiency** | **CpGs > 10X** |
| --- | --- | --- | --- | --- | --- |
| **10083** | L/BD | 97,249,804 | 19.45 | 76.80% | 2,730,286 |
| **10085** | L/BD | 81,002,663 | 16.2 | 77.40% | 2,697,834 |
| **10084** | L/BD | 110,228,135 | 22.05 | 77.30% | 2,956,742 |
| **10089** | L/BD | 94,731,241 | 18.95 | 72.70% | 1,561,159 |
| **10090** | L/BD | 88,216,999 | 17.64 | 74.40% | 1,881,299 |
| **10051** | L/BD | 68,899,204 | 13.78 | 77.50% | 3,060,101 |
| **10087** | L/BD | 113,512,952 | 22.7 | 76.10% | 3,325,543 |
| **Average** |  | **93,405,857** | **18.68** | **76.03%** | **2,601,852** |
| **10082** | H/VHD | 115,476,876 | 23.1 | 74.30% | 2,415,348 |
| **10091** | H/VHD | 83,544,130 | 16.71 | 72.40% | 2,593,687 |
| **10062** | H/VHD | 87,197,006 | 17.44 | 78.20% | 3,115,350 |
| **10061** | H/VHD | 89,455,608 | 17.89 | 70.50% | 2,674,298 |
| **10063** | H/VHD | 84,442,355 | 16.89 | 74.70% | 2,114,928 |
| **10065** | H/VHD | 98,394,582 | 19.68 | 74.60% | 2,858,421 |
| **10066** | H/VHD | 101,539,017 | 20.31 | 72.50% | 3,318,000 |
| **10097** | H/VHD | 87,335,402 | 17.47 | 70.20% | 3,223,308 |
| **10098** | H/VHD | 89,051,187 | 17.81 | 71.00% | 2,474,039 |
| **Average** |  | **92,937,351** | **18.59** | **73.16%** | **2,754,153** |
