# Supplementary material for: Alcohol-dose-dependent DNA methylation and expression in the nucleus accumbens identifies coordinated regulation of synaptic genes
Source: Transl Psychiatry. 2017 Jan 10;7(1):e994–. doi: 10.1038/tp.2016.266 (PMC5545731; doi:10.1038/tp.2016.266)
Supplement: Supplementary Table 2 [file tp2016266x2.doc]

**Supplementary table 2**. Summary of the primers used for bisulfite amplicon sequencing (BSAS).

| **DMR-gene associated** | **Gene accession number** | **F sequence (5')** | **R sequence (5')** | **Amplicon size (bp)** |
| --- | --- | --- | --- | --- |
| *ARHGEF7* | ENSMMUG00000013138 | GTATTTTTTTAATGATGTAGTGAATGAAGTGTTTGTG | ACAACCACAACTCCATCCCCCCAAC | 286 |
| *CDH5* | ENSMMUG00000023525 | AGATCCTGGTATCAGAGGAC | TGGCACACTCGATTTTAGGG | 111 |
| *JAKMIP1* | ENSMMUG00000015099 | TAAATTTTTTTGATAAATGGGGAAATTGTTTTTTGAG | CCTACRACTACAACAAAAAATCCTAAAACTC | 397 |
| *KIRREL3* | ENSMMUG00000006931 | GTAGTGGYGTTGATTTTTTGTTTTTTAGTAAGG | ACACACACACACACACACACCAACCAC | 311 |
| *GPR39* | ENSMMUG00000016115 | TGTAGGTGTTAATGAAAAGTTAGAAGGG | ACCCAAAAACCCAAAACAATACCCTCATTC | 292 |
| *NTM* | ENSMMUG00000020197 | GGGGAGTYGGGGTTTTGAAATTTTAAAAAG | ATTTAATACRAAAAAAACAAACTAAATACCCC | 332 |
| *LRP5* | ENSMMUG00000031237 | AAATTTAYGGTTTATGGTTGAGAATAGTAG | ACRAAAAAAACAAAAAAAACCACTATAAAATC | 311 |
| NBEA | ENSMMUG00000006420 | GATGAGTGTGYGTGTGTATATGTTAGAAGAAGTTG | AAAAAAAACCRCTTTACCAAATCTTTTCAAAATATC | 328 |
