# Supplementary material for: Alcohol-dose-dependent DNA methylation and expression in the nucleus accumbens identifies coordinated regulation of synaptic genes
Source: Transl Psychiatry. 2017 Jan 10;7(1):e994–. doi: 10.1038/tp.2016.266 (PMC5545731; doi:10.1038/tp.2016.266)
Supplement: Supplementary Table 3 [file tp2016266x3.doc]

**Supplementary table 3**. Summary of the primers used to detect the different transcript variants per gene analyzed by real time PCR.

| **DMR-gene associated** | **TV detected (rhesus macaque ID)** | **F sequence (5')** | **R sequence (5')** | **Amplicon size (bp)** |  |
| --- | --- | --- | --- | --- | --- |
| *GPR39* | 201 (**ENSMMUT00000022658**) | GATGACGCACAGGATTTGCG | GGAAGAGCCGTCCATTTGGA | 89 |  |
| *LRP5* | 201 (ENSMMUT00000044668) | AGATCCTGGTATCAGAGGAC | TGGCACACTCGATTTTAGGG | 111 |  |
| *JAKMIP1* | 201 (**ENSMMUT00000042440**) 202 (**ENSMMUT00000021159**) 203 (**ENSMMUT00000042439)** | GAGCGAGATGTGAGGCGATT | GGTCTCTCGCGAGCGTTTCA | 126 |  |
|  | 201 (**ENSMMUT00000042440**) | TGCGCTCGCTACGCCCG | GGAAACCACCATCACTTGGGA | 127 |  |
|  | 202 (**ENSMMUT00000021159**) 203 (**ENSMMUT00000042439)** | GACAGTGGAGGCAAGGAGTG | TACCAGCTCCACCGTGCTAA | 74 |  |
| *PGK1* | 201 (ENSMMUT00000047930)  202 (ENSMMUT00000018061) | GCTGGACAAGCTGGATGTTA | GGCTCCATTGTCCAAGCAGA | 142 |  |
| *BACTIN* | 201  (ENSMMUT00000013155) | GGCTCTCTTCCAACCTTCCT | AGCACTGTGTTGGCGTACAG | 120 |  |
| *TUBA1* | 201  (ENSMMUT00000029653) | TACCTCGACTCTTAGCTTGTCG | TGGATGGAGATGCACTCACG | 107 |  |
|  | | | | | |
