# Supplementary material for: Alcohol-dose-dependent DNA methylation and expression in the nucleus accumbens identifies coordinated regulation of synaptic genes
Source: Transl Psychiatry. 2017 Jan 10;7(1):e994–. doi: 10.1038/tp.2016.266 (PMC5545731; doi:10.1038/tp.2016.266)
Supplement: Supplementary Table 4 [file tp2016266x4.docx]

**Supplementary table 4**. Comparison of the average methylation of 8 DMRs between GWBS and BSAS.

| **DMR-gene** | **GWBS** | | **BSAS** | | **Correlation meth. vs ethanol** | |
| --- | --- | --- | --- | --- | --- | --- |
|  | **Ave meth. Δ (%)** | **Sidak p-value** | **Ave meth. Δ (%)** | **t-test p-value** | **r** | **p-value** |
| *ARHGEF7* | 28.39 | 8.60E-04 | 7.05 | 0.03 | 0.170 | 0.450 |
| *CDH5* | 10.08 | 5.95E-04 | 3.84 | 0.018 | 0.526 | 0.014 |
| *JAKMIP1* | 17 | 3.30E-03 | 11.91 | 0.003 | 0.408 | 0.038 |
| *KIRREL3* | 10.89 | 4.17E-03 | 11.62 | 0.035 | 0.532 | 0.016 |
| *GPR39* | 4.94 | 1.10E-02 | 2.01 | 0.000103 | 0.416 | 0.039 |
| *NTM* | -16.26 | 1.71E-02 | -7.87 | 0.038 | 0.504 | 0.012 |
| *LRP5* | -12.83 | 1.94E-02 | -10.05 | 0.006 | 0.569 | 0.002 |
| *NBEA* | 9.1 | 3.56E-02 | 4.44 | 0.024 | 0.597 | 0.003 |

**Δ**, is the difference in average methylation between L/BD and H/VHD. GWBS indicates genome-wide bisulfite sequencing; BSAS is bisulfite amplicon sequencing. Corrected Sidak p-value and t-test p-value for the DMR are shown. The Pearson’s correlation coefficient and p-value between DMR average methylation level obtained after BSAS and the average g/kg/day ethanol consumed are also shown.
