# Supplementary figures and images for: Alcohol-dose-dependent DNA methylation and expression in the nucleus accumbens identifies coordinated regulation of synaptic genes
Source: Transl Psychiatry. 2017 Jan 10;7(1):e994–. doi: 10.1038/tp.2016.266 (PMC5545731; doi:10.1038/tp.2016.266)

**a**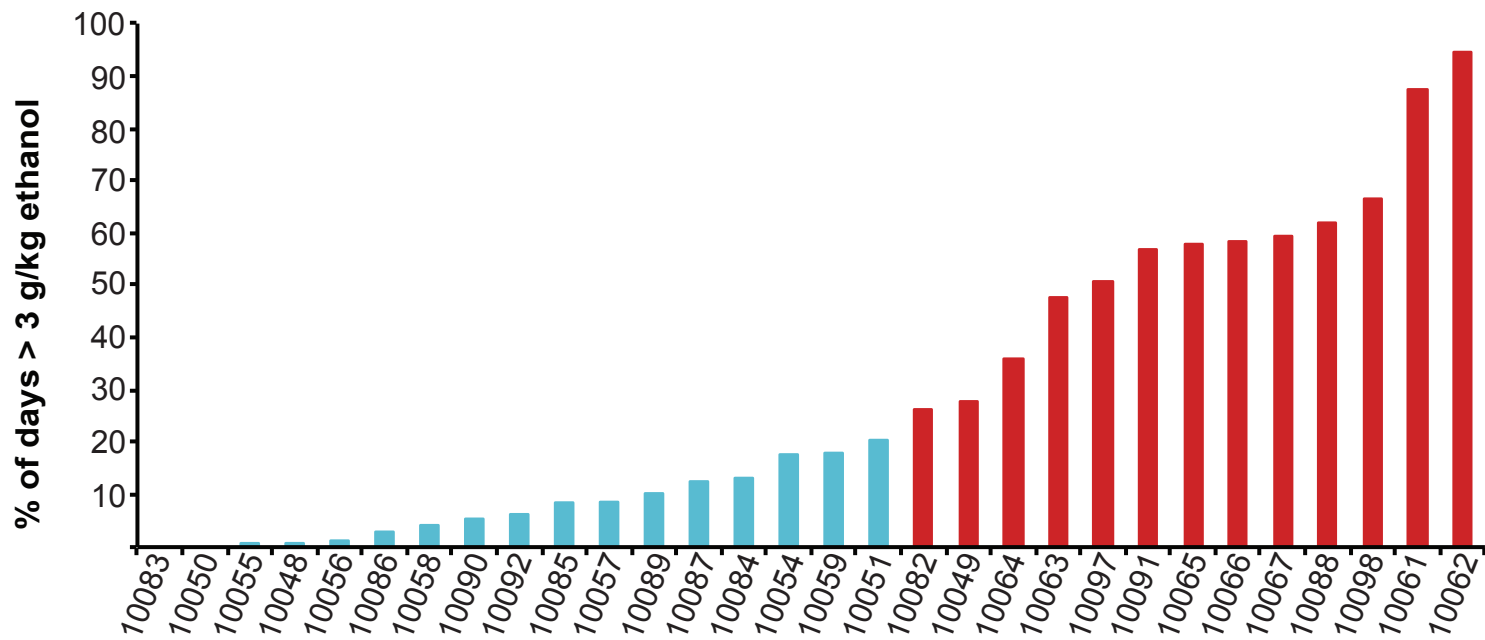**b**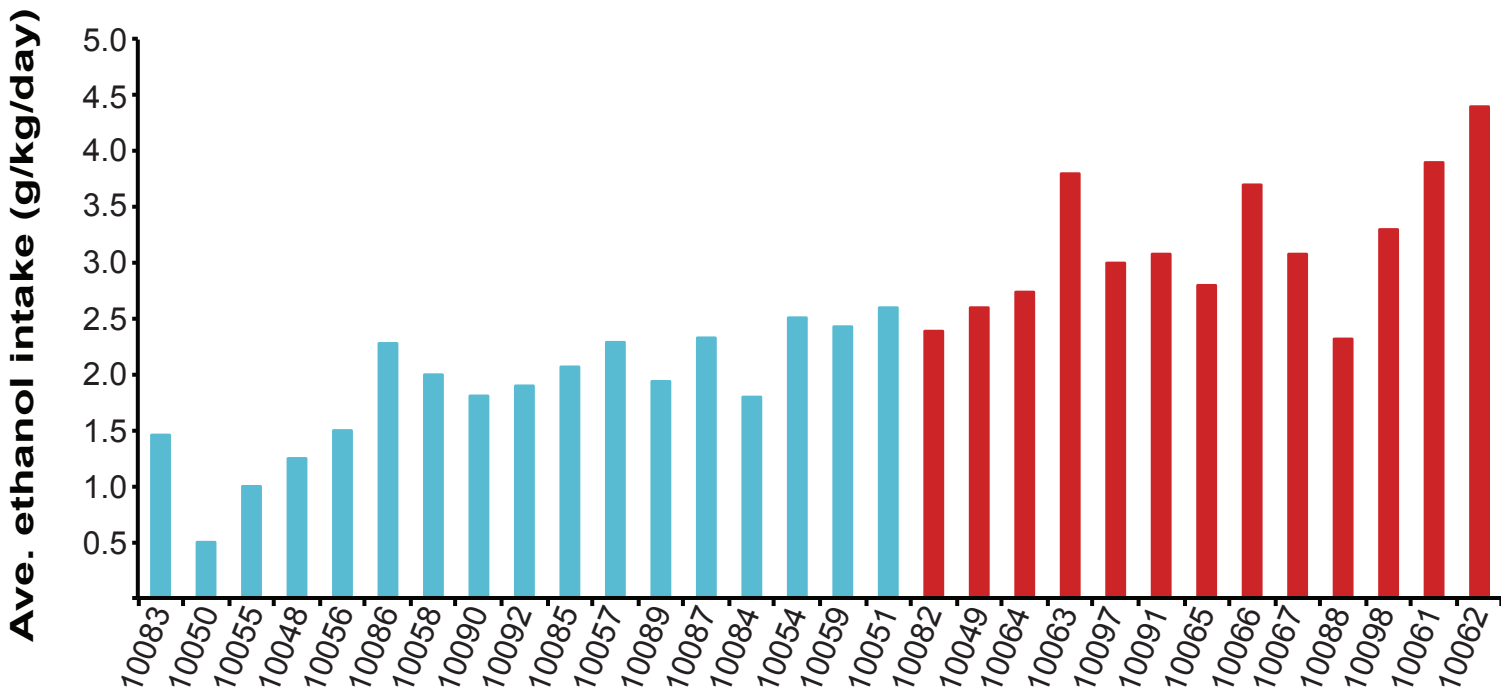

Supplement: Supplementary Figure 1 [file tp2016266x5.pdf]

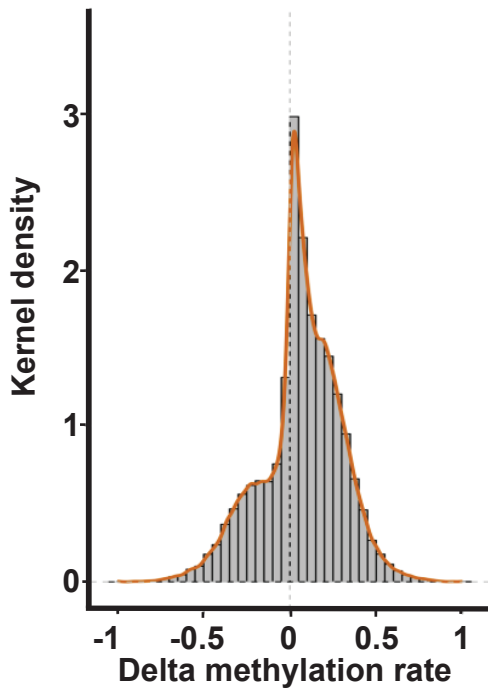

Supplement: Supplementary Figure 2 [file tp2016266x6.pdf]
