# Supplementary material for: Alcohol-dose-dependent DNA methylation and expression in the nucleus accumbens identifies coordinated regulation of synaptic genes
Source: Transl Psychiatry. 2017 Jan 10;7(1):e994–. doi: 10.1038/tp.2016.266 (PMC5545731; doi:10.1038/tp.2016.266)
Supplement: Supplementary Figure Legends [file tp2016266x7.docx]

**Supplementary figure 1**. Classification of the subjects based on their ethanol consumption during 12 months of open access. Subjects were classified as low-binge drinkers (L/BD, blue bars) or heavy drinkers (H/VHD, red bars). **a)** Animals are ranked based on the percent of days consuming more than 3g/kg of ethanol. **b)** The average daily amount of ethanol consumed (g/kg/day) over 12 months is shown.

**Supplementary figure 2**. Kernnel density plot of the average methylation rate of the significant CpGs between L/BD and H/VHD.
